# BTLA inhibition has a dominant role in the *cis*-complex of BTLA and HVEM

**DOI:** 10.3389/fimmu.2022.956694

**Published:** 2022-08-23

**Authors:** Claire Battin, Judith Leitner, Petra Waidhofer-Söllner, Katharina Grabmeier-Pfistershammer, Daniel Olive, Peter Steinberger

**Affiliations:** ^1^ Division of Immune Receptors and T Cell Activation, Center for Pathophysiology, Infectiology and Immunology, Medical University of Vienna, Vienna, Austria; ^2^ Institute of Immunology, Center for Pathophysiology, Infectiology and Immunology, Medical University of Vienna, Vienna, Austria; ^3^ Team Immunity and Cancer, Centre de Recherche en Cancérologie de Marseille (CRCM), Inserm, U1068; Centre National de la Recherche Scientifique (CNRS), UMR7258; Institut Paoli-Calmettes, Aix-Marseille University, Marseille, France

**Keywords:** BTLA, HVEM/TNFRSF14, T cell inhibition, human T cell costimulation, CD160, LIGHT

## Abstract

The engagement of the herpesvirus entry mediator (HVEM, TNFRSF14) by the B and T lymphocyte attenuator (BTLA) represents a unique interaction between an activating receptor of the TNFR-superfamily and an inhibitory receptor of the Ig-superfamily. BTLA and HVEM have both been implicated in the regulation of human T cell responses, but their role is complex and incompletely understood. Here, we have used T cell reporter systems to dissect the complex interplay of HVEM with BTLA and its additional ligands LIGHT and CD160. Co-expression with LIGHT or CD160, but not with BTLA, induced strong constitutive signaling *via* HVEM. In line with earlier reports, we observed that *in cis* interaction of BTLA and HVEM prevented HVEM co-stimulation by ligands on surrounding cells. Intriguingly, our data indicate that BTLA mediated inhibition is not impaired in this heterodimeric complex, suggesting a dominant role of BTLA co-inhibition. Stimulation of primary human T cells in presence of HVEM ligands indicated a weak costimulatory capacity of HVEM potentially owed to its *in cis* engagement by BTLA. Furthermore, experiments with T cell reporter cells and primary T cells demonstrate that HVEM antibodies can augment T cell responses by concomitantly acting as checkpoint inhibitors and co-stimulation agonists.

## Introduction

T cell activation is initiated by antigen recognition *via* the TCR complex and regulated by signals generated by co-stimulatory and co-inhibitory receptors. These so-called immune checkpoints represent attractive therapeutic targets in attempts to manipulate immune responses in autoimmune diseases, virus infections or tumors. Immune checkpoint blockade (ICB) targeting the inhibitory receptors CTLA-4, PD-1 and PD-L1, the major PD-1 ligand, have revolutionized cancer therapy (1, 2). However, only a subset of cancer patients shows a response to current ICB approaches and acquired resistance is frequently observed (3, 4). This has sparked the interest to develop immune checkpoint inhibitors to additional co-inhibitory receptors expressed in T cells such as TIM-3, LAG-3 or BTLA. However, as previously pointed out by us, each of these receptors has unique features and more importantly there are still considerable ambiguities and gaps in our knowledge regarding the functions of these molecules in T cell activation processes (5). Despite the lack of a comprehensive knowledge in relation to the function of these receptors, several antibodies targeting TIM-3 and LAG-3 have been moved into clinical evaluation (6, 7).

BTLA is a member of the CD28 superfamily that exerts inhibitory functions by recruiting SHP-1 and to a lesser extent SHP-2 upon phosphorylation of an immunoreceptor tyrosine-based inhibitory motif (ITIM) and an immunoreceptor tyrosine-based switch motif (ITSM) in its cytoplasmic tail (8). This receptor was shown to be broadly expressed on peripheral human T cells and importantly upon *ex vivo* analysis also on tumor antigen specific CD8^+^ T cells (9). Moreover, an increase of tumor-specific T cell responses upon BTLA-blockade has been reported (9, 10). However, current immune checkpoint inhibitors targeting BTLA are not evaluated in clinical studies. This might be owed at least in part to the complex and incompletely understood interaction of BTLA with its ligand, the herpes virus entry mediator (HVEM; TNFRSF14), which is remarkable in several ways: It was the first interaction reported for a member of the TNFR-SF with a member of the CD28-SF. In addition, it forms an interaction between an inhibitory and an activating receptor, which could potentially generate immune potentiating and immune suppressing signals at the same time. In contrast to other co-inhibitory molecules like PD-1, BTLA is expressed on resting T cells and consequently forms a *cis*-complex with HVEM (11). The *cis*-heterodimeric HVEM-BTLA arrangement prevents *in trans* signaling by HVEM ligands thereby suggesting the maintenance of T cells in a resting state (11). However, there is still insufficient knowledge how the co-expression of these receptors impacts T cell activation. Finally, whereas HVEM (apart from its herpes virus ortholog hUL144) is the sole ligand of BTLA, HVEM is part of a complex network that includes conventional ligands belonging to the TNF-SF such as LIGHT and lymphotoxin α, which binds other TNFR-SF members in addition to HVEM. HVEM binds also to virion glycoprotein D of herpes simplex virus (HSV) and to CD160, another member of the Ig-superfamily expressed on T cells. The role of CD160 in immunity is unclear since immune activating as well as inhibitory functions have been ascribed to this receptor (12–14). Recently, the neuron specific SALM5 was identified as another binding partner of HVEM and this interaction was implicated to exert suppressive functions in the central nervous system (15).

It has been shown that tumor-specific T cells in melanoma patients express high levels of BTLA, and that immunotherapies associated with BTLA-downregulation can improve T cell responses (9). Nevertheless, many aspects of the *in cis* and *in trans* signaling network of HVEM-BTLA in the regulation of T cell responses by antigen-presenting cells is still unknown. In the present study, we confirmed extensive co-expression of HVEM and BTLA on naïve as well as *in vitro* activated human T cells. Furthermore, we used a T cell-based reporter platform to dissect the complex relationship between HVEM and BTLA, as well as additional HVEM ligands, *in trans* and *in cis*. We demonstrate that there is a specific interaction between HVEM and BTLA *in cis* that dramatically alters HVEM signaling in T cell reporters and potentially also in primary CD4^+^ and CD8^+^ T cells. Furthermore, our result indicated that agonistic HVEM antibodies could have utility in immunotherapy approaches since they are capable to block BTLA-mediated inhibition while maintaining activating HVEM signaling in T cells and other immune cells. In comparison to other co-stimulatory molecules like 4-1BB or inhibitory receptors such as PD-1, the HVEM/BTLA axis represents a challenging target, but offers the unique modality namely deploying antibody drugs that function both as co-stimulation agonists and as immune checkpoint inhibitors.

## Material and methods

### Cell culture, antibodies and flow cytometry

The Jurkat cell line (JE6.1) and the mouse thymoma cell line BW5147 were derived from in house stocks. For transfection, HEK293T cells were used. All cells used within this study were cultured in RPMI1640 supplemented with 10% FBS, penicillin (100 U/mL) and streptomycin (100 μg/mL) (from Sigma Aldrich, St. Louis, MO). Mycoplasma detection was monitored using a recently described reporter system based on a human monocytic THP-1 cell line (16). Jurkat reporter cells expressing BTLA, BTLA-Δcyt, HVEM, CD160, LIGHT as well as TCS expressing CD86, BTLA, HVEM, CD160, LIGHT, mouse HVEM (mHVEM) and ICOSL were generated by retroviral and lentiviral transductions. TPR cells expressing BTLA, HVEM, 4-1BB, CD160-GPI, CD160-TM and chimeric mICOS receptors were also generated by retroviral and lentiviral transductions. Surface expression was confirmed by flow cytometry. The following antibodies from Biolegend (San Diego, CA) were used: PE-BTLA (MIH26), APC-BTLA (MIH26), PE-HVEM (122), PE-CD86 (IT2.2), PE-CD83 (HB15e), PE-CD160 (BY55), APC-CD160 (BY55), PE-LIGHT (T5-39), APC-LIGHT (T5-39), PE-mHVEM (HMHV-1B18), APC-CD14 (63D3), FITC-CD56 (HCD56), BV421-CD19 (HIB19), CD4-BV421 (OKT4), CD8-PerCP (HIT8α) and CD25-PeCy7 (M-A251). Surface expression of mICOS:chimera was assessed using an human/mouse ICOS-APC antibody (C398.4A, Biolegend). HVEM antibody (clone SL030717) was described previously (17). HVEM antibody AF356 (polyclonal goat IgG) and CD160 antibody (clone 688327) were purchased from R&D systems (Minneapolis, MN). Binding of the CD160 antibody 688327 was detected with PE- labelled goat-anti mouse-IgG (Fc specific) antibodies (Jackson ImmunoResearch, West Grove, PA, USA). BTLA antibody (clone 6F4) was obtained from Adipogen (Life Sciences, San Diego, CA). An APC-conjugated antibody to mouse CD45 (#104, Biolegend) was used to exclude TCS from Jurkat cells in reporter assays.

For blocking assays Jurkat-NFκB::eGFP control and HVEM expressing cells were incubated with the polyclonal HVEM Ab AF356 (final 10 μg/ml) for 20 minutes at 4°C. After a washing step, BTLA-Fc or LIGHT-Fc fusionprotein (final 3 μg/ml) was added. Binding was detected with goat-anti human IgG-Fcγ antibodies ((Jackson ImmunoResearch). For flow cytometry-based quantification of HVEM and BTLA expression, HVEM and BTLA were stained by PE-HVEM and PE-BTLA mAb and their expression levels were quantified using QUANTUM-R-PE MESF kit (Bangs Laboratories Inc.) according to the manufacturers’ instructions. A comprehensive list of the antibodies and fusionproteins used in this study is provided in **Supplementary Table 1**.

Flow cytometry was performed on an LSRFortessa or on a FACSCalibur flow cytometer (Becton Dickinson Immunocytometry System, San Jose, CA), using FACSDiva and CellQuest software, respectively. Data was analysed with FlowJo (version 10.6.1, Tree Star, Ashland, OR) and Graphpad Prism (version 5, GraphPad Software, Inc., La Jolla, CA). BioRender was used for graphical abstract. Cells were sorted with the SH800S Cell Sorter (Sony Biotechnology, San Jose, CA).

### Generation of cell lines

The generation of the Jurkat NFκB::eGFP reporter cell line as well as the triple parameter (TPR, based on the Jurkat cell line; JE6.1) cell line has been described previously (18, 19). Jurkat NFκB::eGFP cells or TPR were retrovirally or lentivirally transduced to express HVEM, BTLA, BTLAΔcyt, CD160 (CD160-GPI isoform 1 and CD160-TM isoform 3 of UniProt O95971), LIGHT, 4-1BB or mICOS chimeric receptors. BTLAΔcyt harbors 17 amino acids of PD-1 in the intracellular domain (aa 1-178 of BTLA of Uniprot Q7Z6A9; aa 192–208 of microsociety PD-1 of UniProt Q15116-1). The mICOS chimeric constructs consist of a mICOS extracellular domain (aa 1–144 of UniProt Q9WVS0-1) followed by a codon optimized CD28 transmembrane domain (aa 153–179 of UniProt P10747) and fused to the intracellular domain of CD28 (aa 183-220 of UniProt P10847), BTLA (aa 179-289 of Uniprot Q7Z6A9) or CD160 (aa 182-234 of CD160-TM (isoform 3) of Uniprot O95971). As non-signalling control, mICOSΔcyt was used. The mICOSΔcyt molecule that was used harbored the membrane proximal amino acids of PD-1 (aa 192–208 of UniProt Q15116-1), which are not involved in signal-transduction but mediate higher expression of the truncated molecule. Cells that were lentivirally transduced with the pHR‐SIN‐BX_IRES-Emerald are put under selection with puromcyin. Jurkat NFκB::eGFP cells HVEM/BTLA^low^ and HVEM/BTLA^high^ were sorted for high and low expression of BTLA. In addition, Jurkat NFκB::eGFP HVEM cells were transduced with BTLA, BTLA-Δcyt, LIGHT or CD160 and sorted for the respective receptor/ligand. T cell stimulator cells (short: TCS), based on the BW5147 cell line, were engineered as previously described (20, 21). Briefly, BW cells were transduced to stably express an anti-human CD3 single chain fragment fused to a human CD14 stem (CD5L-OKT3scFv-CD14). TCS were retrovirally transduced with HVEM, BTLA, CD86, murine HVEM (mHVEM), LIGHT, CD160 and mICOSL, which was followed by generation of single cell clones for high cell surface expression levels. Cell surface expression of all molecules was further confirmed *via* flow cytometric analysis. FlowJo software (version 10.4.1, Tree Star, Ashland, OR) was used for flow cytometry data analysis.

### Reporter assays

Reporter cells (5 x 10^4^ cells/well) were co-cultivated with TCS (2 x 10^4^ cells/well) for 24 hours at 37°C with 5% CO_2_ in 96 well flat bottom plate. In the case of blocking experiments, Jurkat and TCS were co-cultured in the presence or absence of HVEM antibodies (monoclonal #SL030717 or polyclonal goat IgG #AF356) or a BTLA antibody (clone 6F4) at different concentrations. Subsequently, cells were harvested and stained with an APC-conjugated mCD45.2 antibody. Reporter genes expression of mono- or triple parameter reporter cells (eGFP, eCFP and mCherry) were then analysed *via* flow cytometry using a FACSCalibur or LSRFortessa™ flow cytometer, as previously described (18, 22). Geometric mean of fluorescence intensity (gMFI) of viable reporter cells (APC-negative) was used for further analysis. Fold induction (gMFI) was calculated as follows: reporter gene induction in response to stimulation with co-ligands (stimulation with TCS-ligand) normalized to reporter gene expression of cells stimulated with control TCS.

### Proliferation assays with primary PBMCs

For cell surface expression of receptors, PBMCs isolated from healthy donors were labelled with CFSE as previously described (23). CFSE-labelled PBMCs were either left unstimulated or stimulated with SEE (Staphylococcal enterotoxin E, Toxin Technology, Sarasota, FL; final concentration: 100 ng/ml). On day 3 and 10 expression of HVEM and BTLA was analysed on CFSE^low^ CD4^+^ and CD8^+^ T cells.

For proliferation assays, CFSE-labelled PBMCs (1 x 10^5^/well) were then co-cultured with mitomycin C treated TCS control, TCS CD86, TCS BTLA, TCS HVEM, TCS CD160, TCS LIGHT and TCS 4-1BBL (2 x 10^4^/well) in 96-well round bottom plates for 5 days. In detail, TCS cells were pre-treated with mitomycin C (final concentration 15 μg/ml, Carl Roth, Karlsruhe, Germany) for 30 min at 37°C. Subsequently, cells were washed three times with 1 x PBS and added to PBMCs. Cells are kept in RPMI1640 supplemented with 10% FBS, penicillin (100 U/mL) and streptomycin (100 μg/mL). A HVEM polyclonal antibody (goat IgG) was pre-incubated (final 5 μg/ml) with PBMCs for 30 min followed by the addition of TCS control and TCS HVEM. After 5 days, cells are harvested and CFSE dilution as well as CD25 expression was analysed on CD4^+^ and CD8^+^ T cells. For each donor the assay was performed in triplicates. A single data point represents one donor. For each experiment, supernatant was harvested and stored at −20°C. Cytokine measurement was performed by Luminex multiplex cytokine analysis (100/200™ System, Luminex Corporation). The concentration of IFN-γ, TNF-α and GM-CSF was measured according to the manufacturer’s instructions.

## Statistics

Statistical calculations were performed using GraphPad Prism (Version 7, GraphPad Software, Inc., La Jolla, CA, USA).

Unless indicated otherwise one-way ANOVA with Dunn’s multiple comparison was performed. Levels of significance were categorized as follows: ns, not significant; p > 0.05; *p ≤ 0.05; **p ≤ 0.01; ***p ≤ 0.001.

## Study approval

The study with peripheral blood mononuclear cells (PBMCs) was approved by the ethics committee of the Medical University of Vienna (ECS 1083/2016). All individuals included provided written informed consent. Heparinized whole blood (leucocytes reduction chambers) of healthy donors was purchased from the general hospital in Vienna, Austria (AKH; blood transfusion department) and PBMCs were isolated by standard density-gradient centrifugation using Lymphoprep solution (Technoclone, Austria).

## Results

### BTLA and HVEM are extensively co-expressed on human T cells

**Figure 1:**
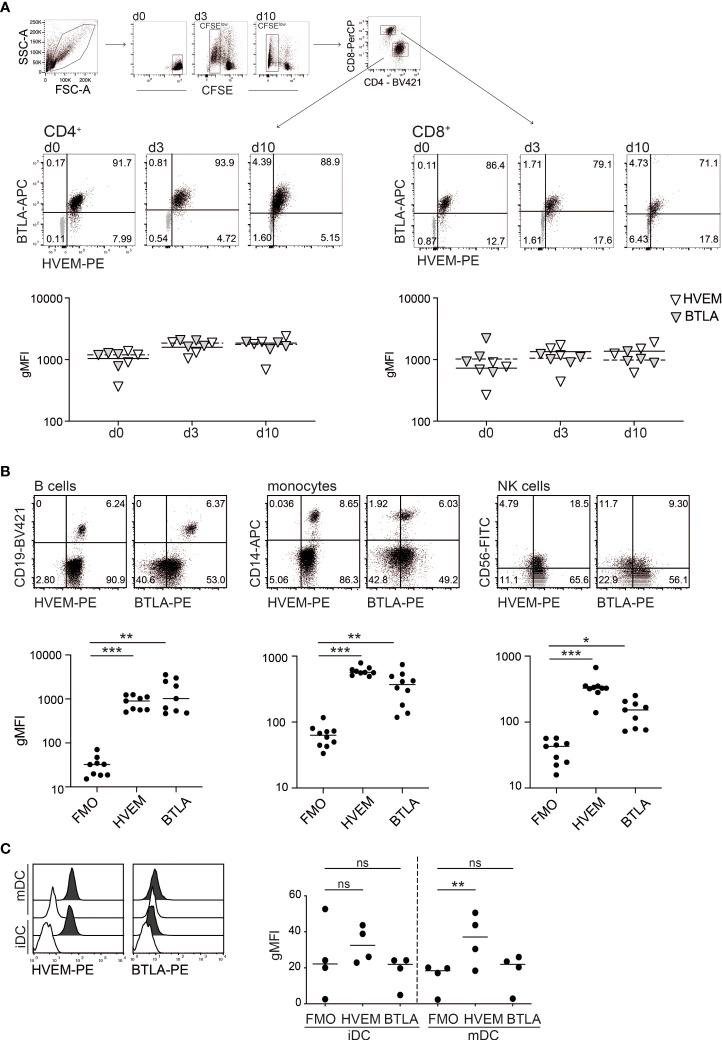
Expression analysis of HVEM and BTLA. **(A)** Gating strategy used to analyse HVEM and BTLA expression on resting (d0) and SEE-stimulated (d3, d10) CFSE-labelled PBMCs derived from healthy donors. Expression was analysed in gated CD4^+^ and CD8^+^ T cells at the indicated time points. Upper panel: data of one representative donor is shown. Isotype controls are shown in grey. Lower panel: summarized data of geometric mean fluorescence intensity (gMFI) of HVEM and BTLA expression in CD4^+^ and CD8^+^ T cells (gated on CFSE^low^ cells on d3 and d10) (n=4, 3 experiments with 1-2 donors). **(B)** B cells (CD19^+^), monocytes (CD14^+^) and NK cells (CD56^+^) were analysed for HVEM and BTLA expression. Upper panel: blots depict data from one representative donor. Lower panel: summarized data (gMFI is shown; n=9, 4 experiments with 2-3 donors). **(C)** HVEM and BTLA expression was analysed on immature (iDC) and mature dendritic cells (mDC) (filled histogram: specific expression of indicated molecule, open histogram: FMO control), (n=4, 3 experiments with 1-2 donors). **(A-C)** Each symbol represents one donor. Lines indicate median gMFI. For statistical evaluation one-way ANOVA followed by Bonferroni correction was performed (***p ≤ 0.001; **p ≤ 0.01; *p ≤ 0.05; ns, p > 0.05).

We analysed the expression of BTLA and HVEM on unstimulated T cells in freshly isolated PBMCs and on T cells activated with SEE *in vitro*. In line with previous reports, we observed that these molecules were extensively co-expressed on resting and *in vitro* activated human CD4^+^ and CD8^+^ T cells (**Figure 1A** **;** **Supplementary Figure 1A**) (11). Furthermore, we found that in freshly isolated PBMCs all B cells co-express HVEM and BTLA. Monocytes uniformly stained positive for HVEM, but only a subset of these cells was expressing BTLA. HVEM was also expressed on the majority of NK cells, whereas BTLA expression was detected in less than 50% of CD56-positive cells (**Figure 1B** **;** **Supplementary Figure 1B**). In addition, HVEM is highly expressed on immature dendritic cells (imDCs) as well as on LPS-activated DCs (mDCs), while this is not the case for the inhibitory receptor BTLA (**Figure 1C**). The expression of LIGHT and CD160, the additional ligands for HVEM, was also analysed. Compared to BTLA, the expression of these molecules was more restricted (**Supplementary Figures 1C-E**). These data indicate that HVEM and BTLA are broadly expressed on PBMCs and furthermore that human T cells largely maintain co-expression of these receptors during *in vitro* activation.

### BTLA and HVEM function as bi-directional switch

**Figure 2:**
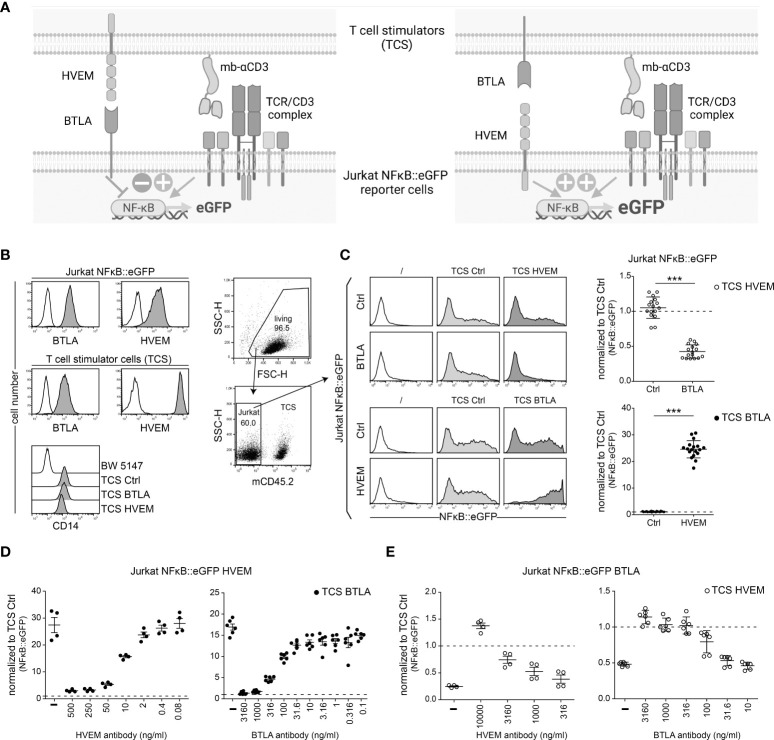
Evaluation of HVEM and BTLA signaling in a reporter cell-based system. **(A)** Schematics illustrating the evaluation of the HVEM/BTLA in Jurkat NFκB::eGFP reporter cells. Left: reporter cells expressing BTLA receive inhibitory signals *via* HVEM expressed on the TCS; Right: reporter cells expressing HVEM receive activating signals *via* BTLA expressed on TCS. **(B)** Expression levels of the indicated cell surface molecules on Jurkat NFκB::eGFP reporter cells and T cell stimulator cells (TCS) analysed *via* flow cytometry. TCS express a membrane-bound anti-human CD3 single chain fragment that is linked to a human CD14 stem (OKT3scFv-CD14). Parental BW5147 cells were used as a control. Open histograms represent control cells whereas grey histograms represent cells expressing the indicated molecules. **(C)** Left panels: Gating strategy used for reporter assays. TCS were excluded by using a mouse CD45.2 antibody and NFκB::eGFP expression was measured *via* flow cytometry. Middle panels: One representative experiment of control Jurkat NFκB::eGFP cells and Jurkat NFκB::eGFP expressing BTLA or HVEM stimulated with control TCS and TCS expressing HVEM or BTLA, respectively, is shown. Histograms of unstimulated reporter cells are also shown. Right panels: The indicated reporter cells were stimulated with TCS HVEM or TCS BTLA and eGFP expression is shown normalized to stimulation with control TCS (dotted line). Results of BTLA and HVEM reporter cells are from 8, respectively 9, independent experiments performed in duplicates. For statistical evaluation, unpaired t-test was performed (***p ≤ 0.001). **(D)** Jurkat NFκB::eGFP cells expressing HVEM were stimulated with TCS BTLA in the absence or presence of a HVEM antibody (clone SL030717) (500, 250, 50, 10, 2, 0.4 and 0.08 ng/ml) or a monoclonal BTLA antibody (clone 6F4) (3160, 1000, 316, 100, 31.6, 10, 3.16, 1, 0.316 and 0.1 ng/ml). **(E)** Jurkat NFκB::eGFP cells expressing BTLA were stimulated with TCS HVEM in the absence or presence of a HVEM antibody (10000, 3160, 1000 and 316 ng/ml) or a BTLA antibody (3160, 1000, 316, 100, 31.6 and 10 ng/ml). **(B-D)** ± SD is shown. Dotted line depicts control stimulation.

The complexity of the HVEM/BTLA/LIGHT/CD160 receptor network and the extensive co-expression of BTLA and HVEM hamper the investigation of these pathways in T cell activation processes. Consequently, we turned to a reductionist T cell reporter system to study how the interplay of these receptors impacts T cell activation. In a first step, we analysed whether BTLA and HVEM signaling can be assessed in a T cell reporter system. We have previously generated a highly sensitive NFκB::eGFP reporter cell line based on Jurkat E6.1 (18). Importantly, these reporter cells lack endogenous expression of HVEM, BTLA as well as LIGHT and CD160 which are also ligands for HVEM (**Supplementary Figure 2**), Consequently, they represent an excellent tool for studying this pathway. The use of the reporter system to study the HVEM/BTLA pathway is illustrated in **Figure 2A**. The Jurkat E6.1 NFκB::eGFP reporter cells were transduced to express human BTLA or HVEM (**Figure 2B**). The reporter cells can be activated with T cell stimulators (TCS), which are cells based on the mouse thymocyte cell line BW5147 that was engineered to express a membrane-bound anti-CD3 antibody fragment (OKT3scFv) that provides signal 1 *via* the TCR-CD3 complex (21). In addition to control TCS that do not express any co-stimulatory or co-inhibitory ligand, TCS were generated that co-express a membrane-bound anti-CD3 antibody and HVEM or BTLA, respectively. Cell surface expression of these molecules on the reporter cells and TCS was confirmed *via* flow cytometry (**Figure 2B** **;** **Supplementary Figure 3**). First, control reporter cells and BTLA-expressing reporter cells were stimulated with control TCS or TCS expressing the corresponding ligand HVEM for 24h. Subsequently, NFκB is measured through the expression of the fluorescent protein eGFP *via* flow cytometry (**Figure 2C**, upper panels). BTLA expressing reporter cells were strongly inhibited upon engagement with the HVEM ligand, whereas it did not affect the activation of control reporter cells (**Figure 2C**). Secondly, to evaluate HVEM activation, HVEM-expressing reporter cells were co-cultured with TCS BTLA and eGFP expression was assessed. We observed a very strong increase in reporter activation when the HVEM receptor interacted with its ligand BTLA. Upon HVEM engagement by BTLA eGFP expression in the reporter cells was partially off scale and therefore the measured gMFI values might not fully reflect the potent stimulatory effect of HVEM (**Figure 2C** lower panels). These effects of reporter inhibition or activation *via* BTLA or HVEM, respectively, were completely and dose-dependently abolished upon the addition of a BTLA or HVEM antibody blocking either the receptor on the reporter cells or the ligand on TCS (**Figures 2D, E** **)**. These experiments demonstrate that BTLA and HVEM function as a bi-directional switch in the activation of T cells. Furthermore, they show that our T cell reporters and stimulator cells represent a platform that is well-suited to study the role of BTLA and HVEM in T cell activation processes.

### HVEM co-stimulation *via* its ligands

**Figure 3:**
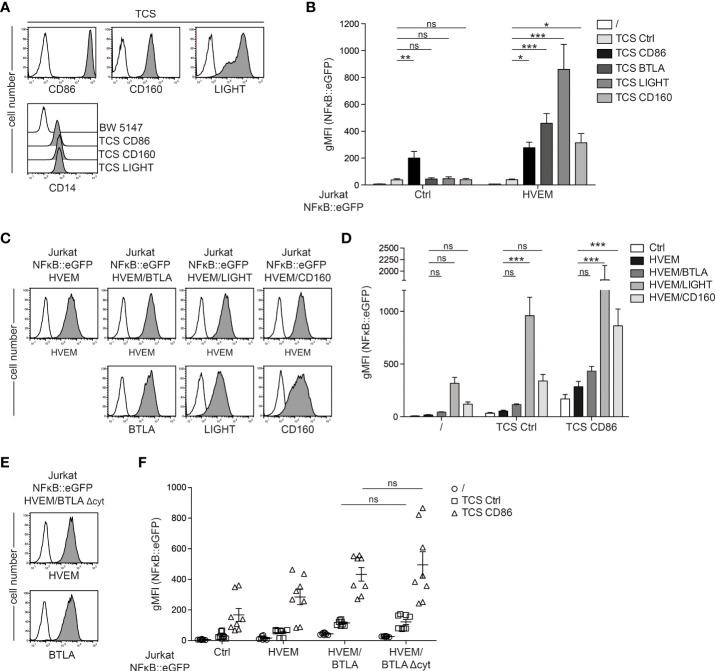
Regulation of HVEM co-stimulation *in trans* and *in cis.* **(A)** Upper panels: cell surface expression of CD86, CD160 and LIGHT on TCS analysed *via* flow cytometry (open histograms: control TCS; grey histograms: TCS expressing the indicated molecules). Lower panel: membrane-bound anti-CD3-fragment (detected *via* its CD14 stem) on TCS analysed *via* flow cytometry. Parental BW5147 cells were used as a control. **(B)** Control reporter cells and HVEM expressing reporter cells were stimulated with control TCS and TCS expressing CD86, BTLA, LIGHT and CD160 for 24 h and NFκB::eGFP expression was measured by flow cytometry. Data is shown from 4 independent experiments performed in duplicates. For statistical evaluation, one-way ANOVA with Dunn’s multiple-comparisons test was performed (***p ≤ 0.001; **p ≤ 0.01; *p < 0.05; ns, p > 0.05). **(C)** Jurkat NFκB::eGFP reporter cells expressing HVEM, HVEM/BTLA, HVEM/LIGHT and HVEM/CD160 were analysed for the expression of the indicated molecules; open histogram show control reporter cells. **(D)** The indicated reporter cells were left unstimulated or were stimulated with control TCS or TCS CD86 and eGFP expression was measured *via* flow cytometry. Results are shown from 4 independent experiments performed in duplicate. For statistical evaluation, two-way ANOVA followed by Bonferroni’s test was performed (***p ≤ 0.001; ns, p > 0.05). **(E)** Expression levels of HVEM and BTLAΔcyt on reporter cells. Open histograms represent control reporter cells. **(F)** The indicated reporter cells were left unstimulated or were co-cultured with control TCS and TCS CD86. eGFP expression was assessed *via* flow cytometry. Data is shown for 4 independent experiments performed in duplicate. For statistical evaluation, two-way ANOVA followed by Bonferroni’s test was performed (***p ≤ 0.001; **p ≤ 0.01; ns, p > 0.05). **(B, D, F)** ± SD is shown.

HVEM does not only interact with BTLA, but also functions as a receptor for the TNF-SF-member LIGHT and CD160, a member of the Ig superfamily. To compare the capability of these ligands to functionally engage HVEM, we additionally generated stimulator cells expressing LIGHT and CD160 (**Figure 3A**). We co-cultured control reporter cells or reporter cells expressing HVEM with control TCS and TCS expressing BTLA, LIGHT or CD160 for 24h and eGFP expression was measured *via* flow cytometry. TCS expressing CD86, which is the ligand for CD28, the primary T cell co-stimulatory receptor, were also used. Expression of CD28 on the reporter cells is shown in **Supplementary Figure 2**. The results of these experiments confirmed that all three ligands strongly co-stimulated T cell reporter cells expressing HVEM. Stimulation *via* CD86-CD28, which is independent of HVEM signaling resulted in similar NFκB activation in control and HVEM-expressing reporter cells (**Figure 3B**). In a next step, we assessed how *in cis* interaction of HVEM with its ligands BTLA, CD160 and LIGHT affects intrinsic HVEM signaling. For this, we co-expressed HVEM reporter cells with BTLA, LIGHT or CD160 and cell surface expression of these molecules was determined *via* flow cytometry (**Figure 3C**). BTLA co-expression had only a modest effect on HVEM activation in our reporter system (**Figure 3D**). In contrast, co-expression of LIGHT and CD160 with HVEM induced very strong NFκB activation in the reporter cells even in the absence of TCR signals, and this effect was further enhanced by stimulation with TCS ctrl and TCS-CD86 (**Figure 3D**). This could indicate that LIGHT and CD160 can functionally engage HVEM *in cis* however it cannot be excluded that HVEM mediated reporter activation is caused by in trans interactions between adjacent reporter cells. To investigate whether the reduced HVEM activation by BTLA *in cis* is due to inhibitory signaling of BTLA, we generated reporter cells co-expressing HVEM and BTLA that lack the cytoplasmic tail (BTLA-Δcyt) (**Figure 3E**). We observe that HVEM/BTLA-Δcyt co-expression does not lead to an increase in HVEM activation (**Figure 3F**). These results indicate that weak reporter activation observed upon co-expression of HVEM and BTLA is due to a low capability of BTLA to functionally engage HVEM *in cis* rather than inhibitory signaling *via* BTLA. In contrast to BTLA, the co-expression of LIGHT or CD160 strongly augments HVEM activation.

### BTLA co-inhibition dominates over HVEM co-stimulation

**Figure 4:**
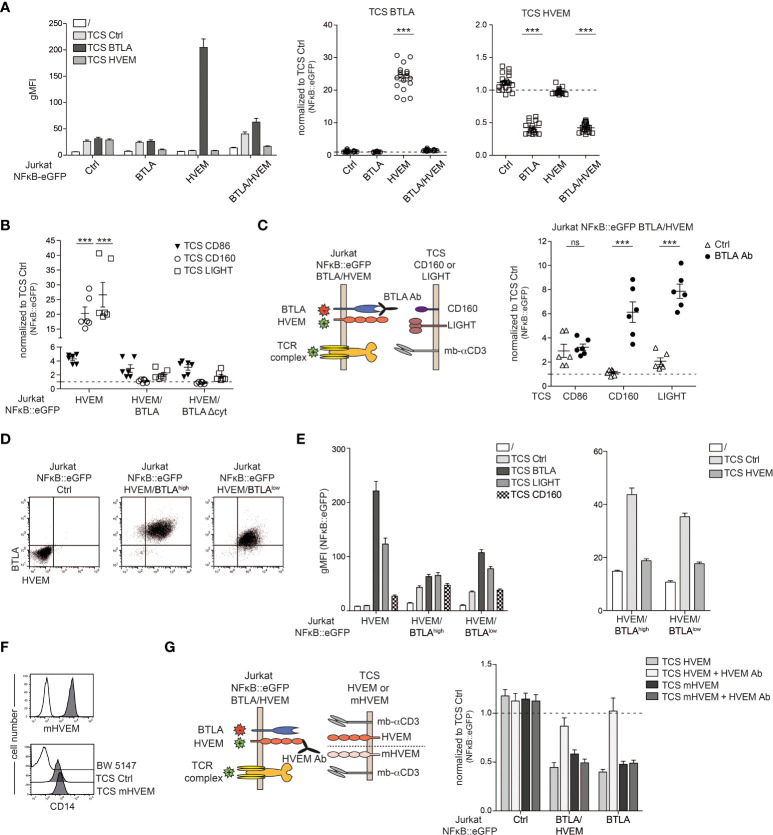
Assessment of co-expression of HVEM/BTLA signaling. **(A)** Control reporter cells and reporter cells expressing BTLA, HVEM or BTLA/HVEM were left unstimulated or stimulated with TCS ctrl, TCS BTLA and TCS HVEM. NF-κB::eGFP activation was measured after 24 h. Left: one representative experiment performed in duplicate is shown. Right: reporter gene expression is shown normalized to reporter gene expression induced by control-TCS (gMFI of reporter cells stimulated with the indicated TCS/gMFI of TCS ctrl stimulated cells). Data is depicted of at least eight independent experiments performed in duplicates. One-way analysis of variance followed by a Dunnett’s multiple comparison test were used for comparison to control reporter cells (***p ≤ 0.001; ns, p > 0.05). **(B)** Jurkat NFκB::eGFP ctrl and Jurkat NFκB::eGFP expressing HVEM, HVEM/BTLA or HVEM/BTLAΔcyt cells were stimulated with TCS ctrl, TCS CD86, TCS CD160 and TCS LIGHT. Reporter gene expression is shown normalized to control-TCS. Results are shown from three independent experiments performed in duplicates. For statistical evaluation, two-way ANOVA followed by Bonferroni’s test was performed (***p ≤ 0.001). **(C)** Left panel: Schematic representation of receptors on reporter cells and ligands on stimulator that were evaluated in absence or presence of a blocking BTLA antibody. Right panel: Reporter cells expressing BTLA/HVEM were stimulated with TCS CD86, TCS CD160 or TCS LIGHT in the presence or absence of a blocking BTLA antibody (5 μg/ml; clone 6F4). Reporter activation induced by the indicated TCS is shown normalized to control-TCS. Data is depicted from three independent experiments performed in duplicate. For statistical evaluation, two-way ANOVA followed by Bonferroni’s test was performed (***p ≤ 0.001; ns, p > 0.05). **(D)** Flow cytometric analysis of cell surface molecules BTLA and HVEM on the indicated reporter cells. **(E)** Left panel: Reporter cells expressing HVEM, HVEM/BTLA^high^ or HVEM/BTLA^low^ were left unstimulated or stimulated with TCS ctrl, TCS BTLA and TCS LIGHT. Right panel: Reporter cells expressing HVEM, HVEM/BTLA^high^ or HVEM/BTLA^low^ were left unstimulated or stimulated with TCS ctrl or TCS HVEM. gMFI of NFκB::eGFP activation is shown. Results are depicted from four independent experiments performed in duplicate. **(F)** Cell surface expression of mHVEM and membrane-bound anti-CD3 on TCS analysed *via* flow cytometry (open histograms: control TCS; grey histograms: expression level of the indicated molecules). **(G)** Left panel: Schematic representation of stimulation experiments of reporter cells co-expressing BTLA and HVEM with TCS expressing human HVEM or mouse HVEM (mHVEM) with and without HVEM antibody SL030717 (10 µg/ml) that blocks human but not mouse HVEM. Right panel: Control reporter cells and reporter cells expressing HVEM/BTLA or BTLA were stimulated with TCS HVEM or TCS mHVEM in the presence or absence of a blocking HVEM antibody. Reporter gene expression is shown normalized to control-TCS. Results are depicted from four independent experiments performed in duplicate. **(A, B, C, G)** ±ΔSD is shown. Dotted line depicts control stimulation.

BTLA and HVEM are co-expressed on the majority of T cells (**Figure 1**) (11). To assess how co-expression of HVEM/BTLA affects engagement of these receptors with their ligands *in trans*, we stimulated HVEM/BTLA reporter cells with TCS expressing HVEM or BTLA. Control Jurkat NFκB::eGFP and Jurkat NFκB::eGFP cells expressing HVEM or BTLA were also included in these experiments. We observed, that compared to reporter cells expressing HVEM alone, HVEM-signaling *via* TCS-BTLA is strongly impaired on BTLA/HVEM reporter cells showing that *in cis* interaction prevents functional engagement of HVEM *in trans*. By contrast, BTLA inhibition upon *in trans* stimulation *via* TCS-HVEM is not affected by co-expression of HVEM. This indicates a functional hierarchy in the cis complex of HVEM and BTLA where HVEM activation by ligands presented *in trans* is abrogated, whereas BTLA mediated inhibition can still occur upon engagement by HVEM *in trans* (**Figure 4A**). In its ectodomain, HVEM has four cysteine-rich domains (CRD) in which distinct ligand binding occurs. While BTLA and CD160 interact with CRD1 on monomeric HVEM, LIGHT engages with CRD2 and CRD3 on trimerized HVEM (14, 24). Therefore, we investigated whether co-stimulation mediated by CD160 and LIGHT would be differentially affected by BTLA-co-expression on HVEM-reporter cells. However, in line with an earlier report the responses to TCS-CD160 and TCS-LIGHT were also greatly reduced in the presence of BTLA (**Figure 4B**) (11). HVEM co-stimulation was also impaired upon co-expression of a BTLA molecule lacking the intracellular signaling domain (BTLAΔcyt) indicating that inhibitory signaling *via* BTLA is not responsible for the reduced response to LIGHT and CD160 (**Figure 4B**). As expected, co-stimulation with TCS-CD86 was not affected by BTLA co-expression. Importantly, HVEM signaling *via* LIGHT and CD160 can be reinstalled by the addition of a BTLA blocking antibody (**Figure 4C**). To follow up on the results summarized in **Figure 4A**, we addressed whether the observed dominance of BTLA inhibition over HVEM co-stimulation was due to an excess of BTLA molecules upon co-expression of these two receptors. To this end, we generated HVEM reporter cells co-expressing BTLA at high and low levels (**Figure 4D**). Stimulation experiments revealed that HVEM co-stimulation is operative to some degree when BTLA is co-expressed only at low levels (**Figure 4E** left panel). However, co-inhibition of BTLA *via* TCS-HVEM is still intact, indicating that signaling of BTLA is not impaired even upon excess of HVEM *in cis* (**Figure 4E** right panel). We have used the Quantum MESF Kit to quantify the number of HVEM and BTLA molecules on the HVEM/BTLA^high^ and the HVEM/BTLA^low^ reporter cells. Compared to HVEM we measured a slightly lower number of BTLA molecules on the HVEM/BTLA^high^ reporter cells and a much lower number of BTLA molecules on the HVEM/BTLA^low^ reporter cells (**Supplementary Figure 4**). Experiments where we selectively blocked HVEM on reporter cells co-expressing HVEM and BTLA with a HVEM antibody (clone SL030717) further corroborated that HVEM-BTLA interaction on T cells does not significantly impair BTLA-mediated inhibition. In these experiments selective blockade of HVEM on the reporter cells was achieved by using TCS expressing murine HVEM, which can functionally engage human BTLA but is not bound by our blocking anti-human HVEM-mAb (**Figure 4F**). Stimulation *via* TCS-mHVEM resulted in inhibition of reporter activation *via* BTLA and this inhibition was not considerably improved by blockade of (human) HVEM on HVEM/BTLA reporter cells (**Figure 4G**). Taken together, our data clearly indicate that in HVEM-BTLA co-expressing T cells HVEM co-stimulation is greatly impaired whereas BTLA mediated co-inhibition is still in place.

### Use of a triple parameter reporter cell line to assess HVEM, BTLA and CD160 signaling

**Figure 5:**
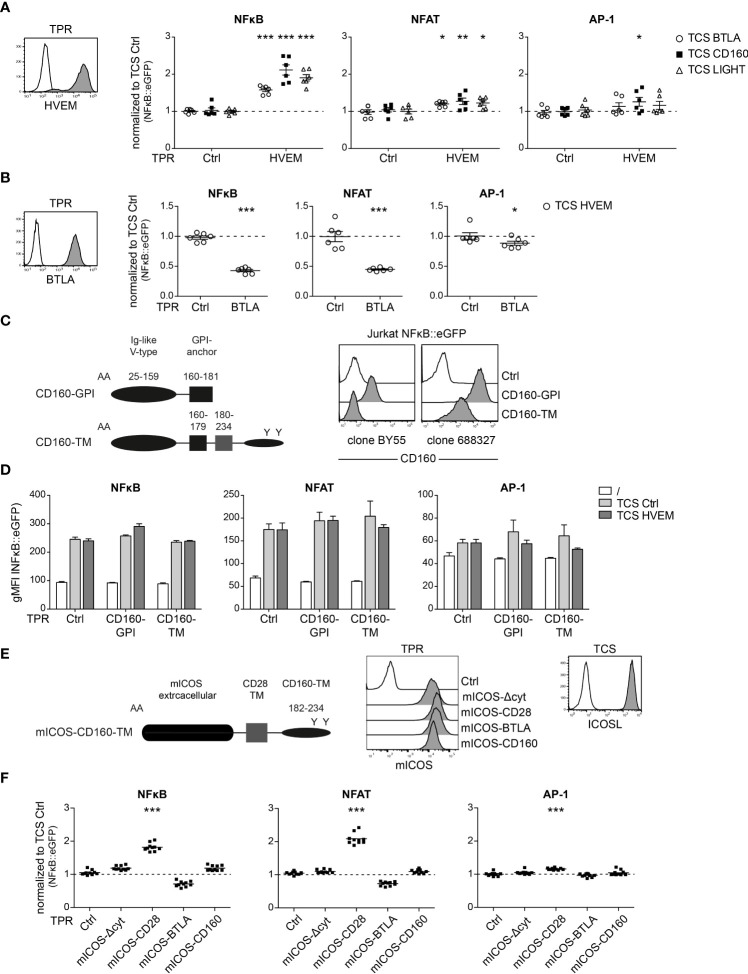
Evaluation of CD160 receptor function in a triple parameter reporter cell system. **(A)** Left panel: HVEM expressing triple parameter reporter cells (TPR; grey histogram) and control-TPR (open histogram) were analysed for HVEM expression. Right panel: Control TPR and TPR expressing HVEM were stimulated with TCS control or TCS expressing BTLA, CD160 or LIGHT. Reporter activation (NFAT::eGFP, NFkB::eCFP and AP-1::mCherry) was assessed *via* flow cytometry. Data of three independent experiments in duplicate is shown. Normalized reporter activation is shown (gMFI reporter gene expression induced by the indicated TCS/gMFI reporter gene expression induced by TCS ctrl stimulated cells). **(B)** Left panel: BTLA expressing triple parameter reporter cells (TPR; grey histogram) and control-TPR (open histogram) were analysed for BTLA expression. Right panel: Control TPR and TPR expressing BTLA were stimulated with control TCS and TCS HVEM. Results are shown from three independent performed experiments in duplicate. Normalized reporter activation is shown (gMFI of TCS HVEM of stimulated cells/gMFI of TCS ctrl stimulated cells). **(A, B)** For statistical evaluation, two-way ANOVA followed by Bonferroni’s test was performed (***p ≤ 0.001; **p ≤ 0.01; *p < 0.05). **(C)** Left panel: Schematic representation of two CD160 isoforms: the glycosylphosphatidylinositol-anchored-CD160 (CD160-GPI) and the transmembrane isoform of CD160 (CD160-TM). Right panel: Cell surface expression of CD160-GPI and CD160-TM on Jurkat TPR cells assessed by using two different antibodies (clone BY55 and clone 688327) (open histogram: control cells; grey histograms: expression of the indicated CD160 molecules). **(D)** Control TPR and TPR expressing the two isoforms CD160-GPI and CD160-TM, respectively, were left unstimulated or stimulated with the indicated TCS. **(E)** Left panel: Schematic representation of a mICOS-CD160-TM chimera. Middle panel: Expression of mICOS chimera on TPR. Right panel: TCS mICOSL (grey histogram) and control TCS (open histogram) were stained with a mICOSL antibody **(F)** mICOS-chimera expressing reporter cells were stimulated with TCS control and TCS mICOSL. Reporter gene expression induced by TCS mICOSL normalized to TCS control. For statistical evaluation, one-way ANOVA with Dunn’s multiple-comparisons test was performed (***p ≤ 0.001; **p ≤ 0.01; *p < 0.05). **(A, B, D, F)** ±ΔSD or mean is shown. Dotted line depicts control stimulation.

CD160 is a cell surface receptor expressed on T cells and NK cells (25). Due to alternative splicing of the *CD160* gene, different isoforms, specifically the GPI-anchored CD160 (CD160-GPI) and a transmembrane version (CD160-TM) are formed (26). The role of the CD160 receptor is still under debate. It has been reported to deliver stimulatory signals by engaging HVEM on NK cells while another study described inhibitory capacities of CD160 in CD4^+^ T cells (12–14). To investigate the role of CD160 as a receptor, we made use of a previously described triple parameter reporter T cell line (TPR), based on the Jurkat T cell line, that allows also to assess the activity of transcription factors NFAT and AP-1, in addition to NFκB. Each of these transcription factors drives the expression of a distinct fluorescent protein (NFκB::eCFP, NFAT::eGFP and AP-1::mCherry), thus allowing to simultaneously analyse the activity of each individual transcription factor. We introduced HVEM, BTLA, CD160-GPI and CD160-TM into these cells and stimulated the reporter cells with stimulator cells expressing the respective ligands (**Figures 5A-D**). HVEM stimulation *via* BTLA, CD160 and LIGHT induced an increase in NFκB activation, while NFAT and AP-1 were only weakly activated (**Figure 5A**). Engagement of BTLA reporter cells with HVEM induced co-inhibitory signaling that resulted in reduced activity of NFκB, NFAT and AP-1 (**Figure 5B**). To investigate the function of CD160, we generated TPR expressing CD160-GPI and CD160-TM. Interestingly, even though these two isoforms do not differ in their extracellular region, the commonly used CD160 antibody (clone BY55) does not detect CD160-TM, while clone 688327 interacts with both forms of CD160 as described earlier (26); (**Figure 5C**). However, BY55 also binds only very weakly to the GPI form and it is therefore possible that BY55 simply fails to react with CD160-TM because of the low expression of this isoform. Control TPR and TPR expressing CD160-GPI or CD160-TM were co-cultured with TCS-ctrl and TCS-HVEM and reporter gene expression was assessed by flow cytometry. Whereas previous studies described co-inhibitory or costimulatory functions for CD160, we did not observe a significant impact on CD160-GPI or CD160-TM signaling upon engagement by HVEM in our T cell reporter system (**Figure 5D**). To further investigate the ability of CD160 to engage intracellular signaling pathways, we generated chimeric constructs where the extracellular region of CD160 is replaced by mouse inducible T cell co-stimulator (mICOS) and the intracellular domain is derived from CD160-TM (amino acids 182-234; Uniprot O95971) (**Figure 5E**). mICOS chimera have been previously used to functionally evaluate the intracellular sequence of TIM-3 (22). As mICOS is not expressed on our reporter cells, chimeric mICOS receptors enable to study the unknown functionality of cytoplasmic domains of receptors *via* engagement with mICOS ligand (mICOSL). As controls, we used mICOS-CD28 and mICOS-BTLA chimera harboring activating and inhibitory signaling domains, respectively. mICOS and mICOSL expression was verified by flow cytometry (**Figure 5E**). A mICOS chimera lacking cytoplasmic sequences (“mICOSΔcyt”) was used as an additional control. Stimulation of mICOS-BTLA and mICOS-CD28 reporter cells with TCS expressing mICOSL resulted in inhibitory and activating signals for all three transcription factors, thereby validating the mICOS-mICOSL system (**Figure 5F**). By contrast, mICOS-CD160 T cell reporter activation upon stimulation with TCS-mICOSL was not significantly enhanced or reduced compared to stimulation with control-TCS (**Figure 5F**). Taken together, our data do not provide evidence that ligand engagement of CD160 mediates inhibitory or stimulatory downstream signaling in our T cell reporter system.

### HVEM engagement in primary T cells

**Figure 6:**
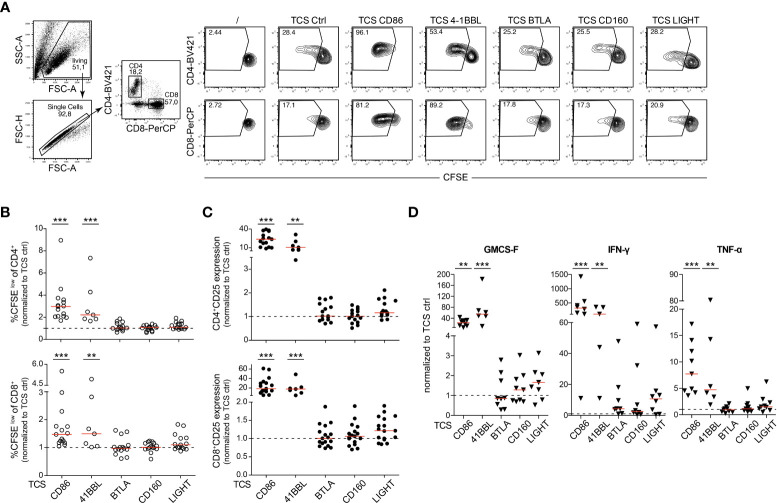
HVEM engagement in primary human T cells. **(A)** Representative contour plots of CFSE-labelled PBMCs stimulated with TCS ctrl, TCS CD86, TCS 4-1BBL, TCS BTLA, TCS HVEM, TCS CD160 and TCS LIGHT for 5 days. Percentages of proliferated (CFSE^low^) of CD4^+^ and CD8^+^ T cells is shown. **(B, C)** CFSE-labelled PBMCs from healthy donors were stimulated for 5 days with the indicated TCS. Proliferation (CFSE^low^) and CD25 upregulation of CD4^+^ and CD8^+^ T cells was measured at day 5. Data is normalized for each donor to stimulation with TCS ctrl. Each data point represents the mean of triplicate measurement of one donor (n = 15; TCS 4-1BBL n = 7). **(D)** Cell culture supernatants of PBMCs stimulated with TCS cells were harvested at day 5 and cytokine expression profile (IFN-γ, GM-CSF and TNF-α) was measured *via* Luminex multiplex cytokine analysis. Each data point represents the mean of triplicate measurement of one donor (IFN-γ: n = 8, TCS 4-1BBL n = 5; GM-CSF and TNF-α: n = 9, TCS 4-1BBL n = 6). **(B-D)** For statistical evaluation, a one-way ANOVA with Dunn’s multiple-comparisons test was performed (***p ≤ 0.001; **p ≤ 0.01). Median is shown (red line). Dotted line depicts control stimulation.

In a next set of experiments, we compared HVEM with the major costimulatory receptors CD28 and 4-1BB (CD137, TNFSF9), another member of the TNFR-SF regarding their capacity to co-stimulate the activation of primary human T cells. 4-1BB is expressed in CD8^+^ T cells, but also in CD4^+^ T cell subsets, B cells and NK cells and mediates activation of these immune cells upon engagement with 4-1BB ligand (4-1BBL) (27). In Jurkat TPR cells the three costimulatory receptors mediated similar induction of NFκB reporter gene expression whereas NFAT activation was only mediated by CD28 as expected (**Supplementary Figure 5**). CFSE-labelled PBMCs isolated from healthy donors were co-cultured with TCS expressing HVEM ligands, 4-1BBL and CD86 (**Figure 6A**). After 5 days of co-culture, proliferation (CFSE^low^) and CD25 expression of CD4^+^ and CD8^+^ T cell subsets and the cytokine concentration in the culture supernatants was analysed. As expected, we observed a strong increase in CFSE^low^ T cells when PBMCs were co-stimulated *via* CD28 or 4-1BB (**Figures 6B, C** **;** **Supplementary Figure 6A**). By contrast co-stimulation of HVEM *via* BTLA and CD160 did not influence the proliferation of CD4^+^ and CD8^+^ T cells and although HVEM stimulation *via* LIGHT slightly increased the proliferation of CD4^+^ and CD8^+^ T cells this trend did not reach statistical significance (**Figures 6B, C** **;** **Supplementary Figure 6A**). Co-stimulation *via* CD28 or 4-1BB, but not *via* HVEM, strongly augmented the upregulation of the activation marker CD25 on CD4^+^ and CD8^+^ T cells (**Figures 6B,C** **;** **Supplementary Figure 6B**). Cytokine production was also not significantly increased by HVEM co-stimulation whereas 4-1BB and CD28 engagement resulted in a dramatic increase of IFN-γ, GM-CSF and TNF-α concentrations in the cell cultures supernatants (**Figure 6D**). Taken together, our data indicate a weak capability of HVEM to co-stimulate the activation of primary human T cells potentially due to its *cis* engagement by co-expressed BTLA.

### HVEM antibodies can have a dual role as immune checkpoint inhibitors and co-stimulation agonists

**Figure 7:**
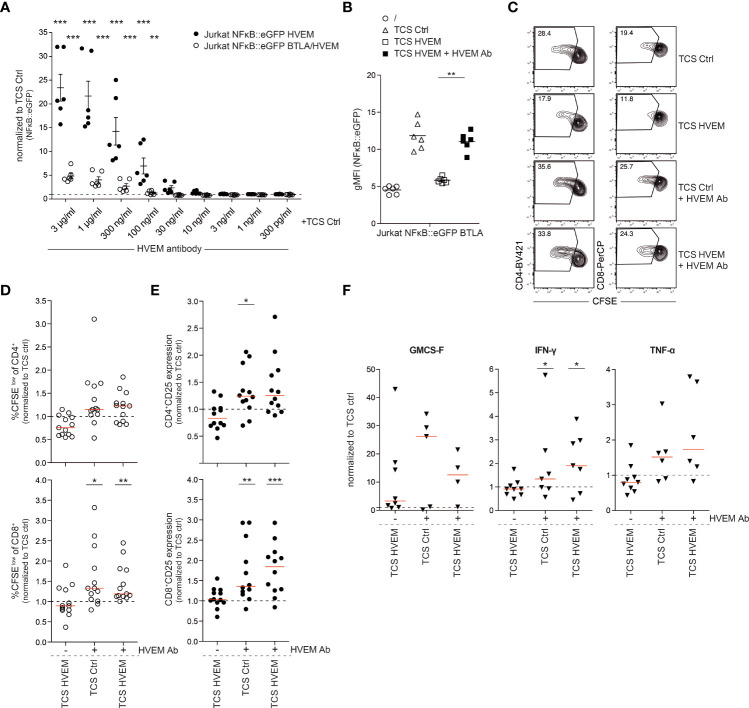
Effect of an HVEM antibody on HVEM co-stimulation. **(A)** HVEM and BTLA/HVEM expressing reporter cells were co-cultured with TCS ctrl in the absence or presence of an HVEM antibody (AF356; R&D systems) at the indicated concentrations. Data of three independent experiments in duplicates is shown. Normalized reporter activation is depicted (gMFI reporter gene expression induced by the indicated TCS or antibody/gMFI reporter gene expression induced by TCS-ctrl stimulated cells). Mean ±ΔSEM is shown. For statistical evaluation, two-way ANOVA followed by Bonferroni’s test was performed (***p ≤ 0.001; **p ≤ 0.01). **(B)** BTLA expressing reporter cells were left unstimulated or were co-cultured with TCS ctrl, TCS HVEM or TCS HVEM in presence of HVEM antibody (AF356). Three experiments in duplicate and median are shown. For statistical evaluation, a one-way ANOVA with Dunn’s multiple-comparisons test was performed (**p ≤ 0.01). **(C)** Representative contour plots of CFSE-labelled PBMCs stimulated with TCS ctrl, TCS ctrl + HVEM antibody and TCS HVEM + HVEM antibody for 5 days. Percentages of proliferated (CFSE^low^) CD4^+^ and CD8^+^ T cells are shown. **(D, E)** CFSE-labelled PBMCs from healthy donors were stimulated for 5 days with the indicated TCS cells. Proliferation (CFSE^low^) and activation (CD25 expression) of gated CD4^+^ and CD8^+^ T cells was measured at day 5. Data is normalized for each donor to stimulation with TCS ctrl. Each data point represents the mean of triplicate measurement of one donor (n = 12). For statistical evaluation, a one-way ANOVA with Dunn’s multiple-comparisons test was performed. **(F)** Cell culture supernatants of PBMCs stimulated with TCS cells were harvested at day 5 and cytokines (IFN-γ, GMCS-F and TNF-α) were measured *via* Luminex multiplex cytokine analysis. Each data point represents one donor in triplicates. For statistical evaluation, a one-way ANOVA with Dunn’s multiple-comparisons test was performed (***p ≤ 0.001; **p ≤ 0.01; *p ≤ 0.05). **(D-F)** ±ΔSD is shown. Dotted line depicts control stimulation.

Antibody-mediated therapy by targeting immune checkpoint molecules has proven to have beneficial effects on cancer patients. Agonistic antibodies to members of the TNFR-SF, such as 4-1BB, CD27, OX40 and GITR, are currently evaluated in cancer patients in numerous clinical trials (28). We identified an HVEM antibody that exerted very potent agonistic effects on HVEM expressing reporter cells. Reporter cells co-expressing HVEM and BTLA were also stimulated albeit at lower levels, indicating that this HVEM agonist is able to partially overcome the inhibition of HVEM mediated by *in cis* interaction with BTLA (**Figure 7A**). This antibody also blocked the interaction between HVEM with BTLA and LIGHT fusion proteins and intriguingly completely reverted HVEM-mediated inhibition of BTLA expressing reporter cells (**Figure 7B** **;** **Supplementary Figure 7**). This indicates that it can concomitantly function as a co-stimulatory agonist by inducing HVEM signaling and as a classical immune checkpoint inhibitor by blocking the engagement of the inhibitory receptor BTLA. This was corroborated in primary human T cells stimulated with control TCS and TCS expressing HVEM which mediate inhibition *via* BTLA. The HVEM antibody augmented proliferation, CD25 expression and cytokine production in CD4^+^ and CD8^+^ T cells stimulated by control TCS as well as TCS expressing HVEM (**Figures 7C-F**, **Supplementary Figures 8A, B**). Importantly, T cells stimulated with TCS HVEM in the presence of this antibody proliferated stronger than T cells stimulated with control TCS indicating a dual role for this antibody as an immune checkpoint inhibitor and a co-stimulatory agonist in primary human T cells.

## Discussion

T cell activation and inhibition is regulated by various co-stimulatory and co-inhibitory molecules. BTLA functions as an inhibitory receptor that dampens immune responses and BTLA^-/-^ mice show increased autoimmune diseases and allergic airway inflammation, as well as deficits in tolerance induction and T cell homeostasis (29–32). Moreover, consistent with an inhibitory role, blockade of BTLA has been shown in several studies to enhance T cell responses *in vitro* (9, 10, 23, 33–35). A recent report on the first genome-wide CRISPRa screen in primary human T cells identified BTLA as a negative regulator of IFN-γ production (36). BTLA restrains T cell help to germinal center B cells and there are several lines of evidence that loss of BTLA signaling promotes lymphoma development (37–40). However, the cytoplasmic domain of BTLA has also been shown to mediate activating signaling pathways and impaired persistence of T cells lacking BTLA upon adoptive transfer was observed (11, 41–43). BTLA signaling is triggered by HVEM, which is part of a complex signaling network that controls both activating and inhibitory responses (44–48).

Here, we have set out to investigate the interplay of BTLA and HVEM in the regulation of human T cell responses. In line with earlier studies, we observed that these two receptors are extensively co-expressed in resting as well as *in vitro* activated T cells. Moreover, both molecules were also present on the surface of APC, such as B cells, and a large subset of monocytes. LIGHT and CD160 expression was more restricted suggesting that BTLA is the major HVEM ligand on human immune cells. The extensive co-expression of both interaction partners and the fact that potentially activating as well as inhibitory signals are generated during engagement of BTLA and HVEM makes it challenging to study this pathway in primary cells. It is of great interest to understand how the *cis*-complex of these two receptors functions in the regulation of T cell responses. Pioneering work by Cheung and colleagues, using a HEK293-based reporter cell line, have shown that functional *in trans* engagement of HVEM is strongly impaired upon *in cis* engagement of HVEM (11). Here, we have used human T cell reporter systems to gain mechanistic insights into the role of these two molecules in T cell activation processes. In initial experiments, we verified that co-engagement of the TCR/CD3 complex with HVEM and BTLA generated potent co-stimulatory and co-inhibitory signals, respectively. In line with Cheung et al., we confirmed that HVEM co-stimulation is strongly inhibited by the presence of BTLA *in cis*. Unexpectedly, however we found that BTLA is still functional in the BTLA/HVEM heterodimeric complex. This has not been previously shown and indicates a functional hierarchy were BTLA co-inhibition dominates over HVEM co-stimulation. This is reminiscent of the cis interaction complex between PD-L1 and CD80 where PD-L1 can no longer engage PD-1 but CD80 can still promote CD28 costimulation (49). Whereas *in cis* interaction of BTLA and HVEM did not result in significant reporter activation, we observed that co-expression of HVEM with CD160 or LIGHT induced strong NFκB activation. Cheung et al. also described potent HVEM mediated reporter activation upon co-expression of gD, another HVEM-ligand. Taken together, this indicates a unique *in cis* engagement of HVEM by BTLA that does not trigger significant HVEM-signaling. By co-expressing HVEM with a BTLA variant lacking a cytoplasmic domain, we ruled out that BTLA-mediated inhibition by HVEM is responsible for this phenomenon, which is in line with earlier reports (11). Altogether our results indicate that BTLA uses two distinct mechanisms to dampen T cell responses: it transmits inhibitory signals upon engagement and in addition it acts as an extrinsic regulator of HVEM co-stimulation.

In order to investigate the impact of HVEM co-stimulation in primary human T cells, we performed co-cultures of PBMCs and T cell stimulator cells expressing different HVEM-ligands. In contrast to the primary costimulatory molecules 4-1BB or CD28, HVEM co-stimulation did not significantly augment proliferation, activation marker (CD25) induction and cytokine production in primary human T cells. These results are in line with an earlier study from us, where we found that T cell stimulators expressing ligands for the TNFR 4-1BB, OX40, CD27 and GITR co-stimulated activation of human T cells *in vitro* whereas the presence of LIGHT did not significantly enhance proliferation and cytokine production (50). Blockade of *in trans* engagement by the HVEM/BTLA complex is likely to play a role in the low costimulatory capacity of HVEM-ligands. Here, we have identified an HVEM antibody that blocks BTLA inhibition and also has potent agonistic properties. Experiments with T cell reporters co-expressing HVEM and BTLA indicate that this antibody is able to partially overcome *in cis* blockade of HVEM by BTLA. Interestingly, we found that this antibody acts, on one hand, by blocking BTLA-mediated inhibition and, on the other hand, by co-stimulating proliferation, CD25 upregulation and cytokine production in primary human T cells. This HVEM antibody can indeed exert a dual function and target the coinhibitory BTLA as an immune checkpoint blocker and the activating receptor HVEM as a co-stimulation agonist.

CD160 is the second HVEM ligand that belongs to the immunoglobulin superfamily. Apart from the major GPI-linked variant, a transmembrane version of CD160 is generated by alternative splicing. Activating as well as inhibitory immune functions have been reported for this receptor (14, 26, 51–54). Thus, CD160 signaling is considered to depend on the cellular context (46). In our study, both CD160 isoforms did not significantly modulate TCR/CD3 signaling upon HVEM engagement in a T cell reporter platform that concomitantly measured the activity of the three transcription factors, which have major roles during T cell activation. Peretz et al. report that blocking the interaction between CD160 and HVEM potently increased the proliferation of CMV and HIV-1 specific CD8^+^ T cells in HIV-1 infected individuals (55). However, the antibody used to block CD160 – HVEM interaction in this study was the same HVEM antibody that we have identified as a potent HVEM agonist. Therefore, it is very likely that the effects observed by Peretz and colleagues are at least in part due to antibody-mediated HVEM co-stimulation.

Taken together, our results highlight the complexity of the BTLA-HVEM pathway that involves the interaction of an activating and an inhibitory receptor and where *cis-*interactions between HVEM and BTLA result in a dominance of BTLA co-inhibition over HVEM co-stimulation. There are a number of reports that indicate that release of BTLA-inhibition can enhance T cell responses *in vitro* and *in vivo*, but deprivation of HVEM signaling upon blockade of BTLA, which is most abundant HVEM ligand in immune cells is a potential drawback of such strategies. This could be overcome by the use of HVEM antibodies that not only function as blockers of BTLA, but also as agonists for HVEM.

## Data availability statement

The original contributions presented in the study are included in the article/**Supplementary Material**. Further inquiries can be directed to the corresponding author.

## Author contributions

CB performed the majority of experiments, supervised experimental work, designed the study and wrote the manuscript. JL performed experiments and designed the study. PW-S performed cytokine measurements. KG-P supervised experimental work. DO provided essential reagents. PS supervised experimental work, designed the study and wrote the manuscript. All authors contributed to the article and approved the submitted version.

## Funding

This study was supported by funds of the Austrian Science Fund (FWF-P32411) to PS.

## Acknowledgments

We wish to thank Claus Wenhardt for technical assistance.

## Conflict of interest

The authors declare that the research was conducted in the absence of any commercial or financial relationships that could be construed as a potential conflict of interest.

## Publisher’s note

All claims expressed in this article are solely those of the authors and do not necessarily represent those of their affiliated organizations, or those of the publisher, the editors and the reviewers. Any product that may be evaluated in this article, or claim that may be made by its manufacturer, is not guaranteed or endorsed by the publisher.
