# Supplementary material for: BTLA inhibition has a dominant role in the cis-complex of BTLA and HVEM
Source: Front Immunol. 2022 Aug 23;13:956694. doi: 10.3389/fimmu.2022.956694 (PMC9446882; doi:10.3389/fimmu.2022.956694)
Supplement: Supplementary file 1 [file DataSheet_1.pdf]

Supplementary information

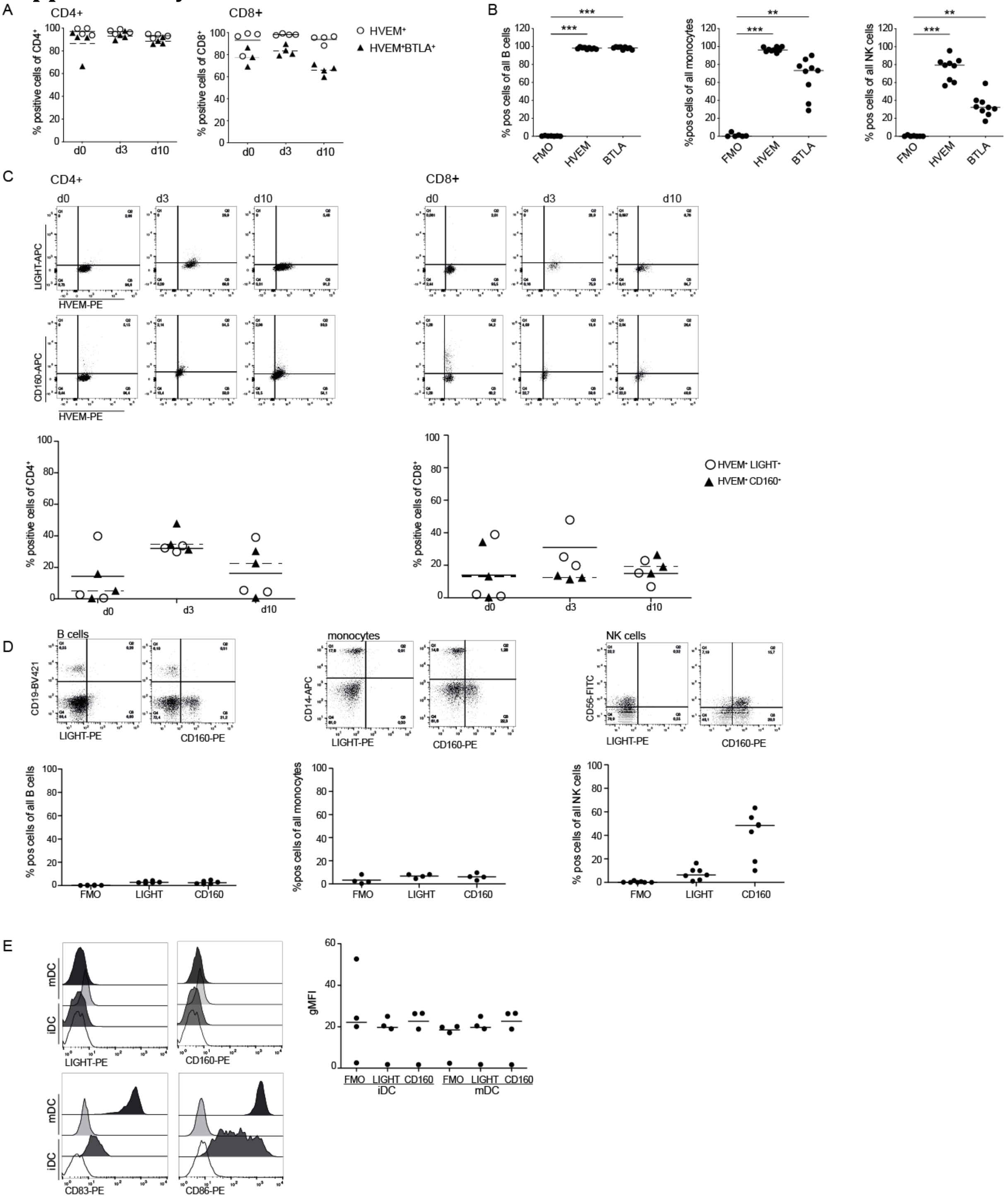

Supplementary Figure 1: Expression of HVEM and BTLA on PBMCs

A) Flow cytometry analysis of HVEM$^+$ and HVEM$^+$/BTLA$^+$ expression on resting (d0) and SEE-stimulated CFSE-labelled PBMCs (d3, d10) derived from healthy donors. Expression of HVEM and BTLA was analysed on CFSE^{low} CD4+ and CD8+ T cells on d3 and d10. Percentage of positive cells is depicted. (n=4, 3 experiments with 1-2 donors) B) B cells (CD19+), monocytes (CD14+) and NK cells (CD56+) were analysed for HVEM and BTLA expression. Percentage of positive cells for HVEM and BTLA is depicted. (n=9, 4 experiments with 2-3 donors). C) Flow cytometry analysis of LIGHT and CD160 expression on resting and SEE-stimulated CFSE-labelled PBMCs. Co-expression of LIGHT and CD160

with HVEM was analysed in CD4$^+$ and CD8$^+$ T cells at the indicated time points. Data of one representative donor is shown. (n=4, 3 experiments with 1-2 donors). D) B cells (CD19$^+$), monocytes (CD14$^+$) and NK cells (CD56$^+$) were analysed for LIGHT and CD160 expression. Upper panel: blots depict data from one representative donor, lower panel: summarized data (n=9, 4 experiments with 2-3 donors) is shown. E) LIGHT and CD160 expression was analysed on immature (iDC) and mature dendritic cells (mDC) (n=4, 3 experiments with 1-2 donors). (A-E) Each symbol represents one donor. Lines indicate median percentage or median gMFI (C). For statistical evaluation one-way ANOVA followed by Bonferroni correction was performed (***p ≤ 0.001; **p ≤ 0.01; ns, p > 0.05).

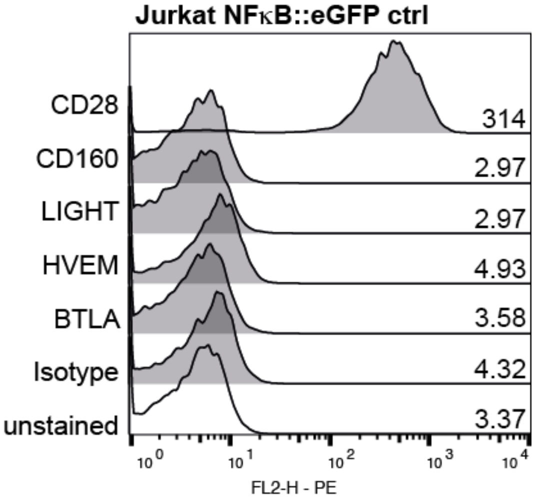

Supplementary Figure 2: Jurkat NF-κB::eGFP reporter cells lack endogenous expression of HVEM, BTLA, LIGHT and CD160. Reporter cells were left unstained (open histogram) or stained with the indicated antibodies (filled histogram). gMFI values are shown.

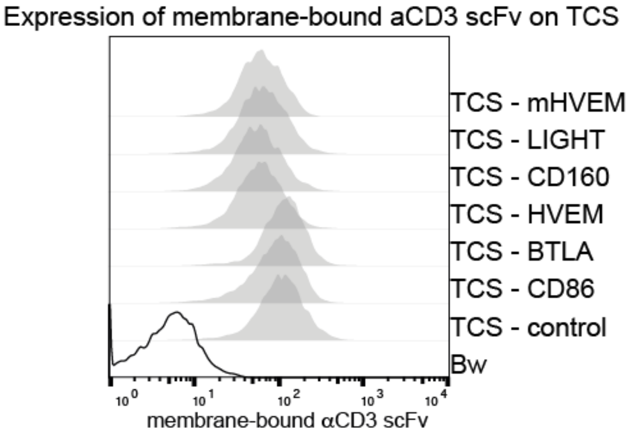

Supplementary Figure 3: T cell stimulator cells were analysed for the expression of the membrane-bound anti-CD3 single chain fragment using a PE - labelled Goat anti-Mouse IgG (H+L)-conjugate (Jackson ImmunoResearch).

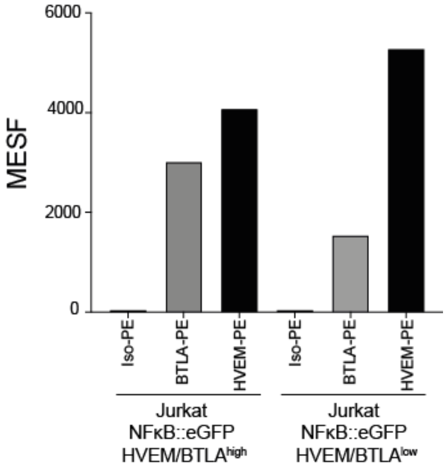

Supplementary Figure 4: **Flow cytometry-based bead-based quantification of HVEM and BTLA expression**. For quantification of HVEM and BTLA expression, HVEM and BTLA were stained on Jurkat reporter cells expressing HVEM and high or low levels of BTLA with PE-HVEM and PE-BTLA mAb. Their expression levels were quantified using QUANTUM-R-PE MESF kit (Bangs Laboratories Inc.) according to the manufacturers' instructions (MESF: Molecules of Equivalent Soluble Fluorochrome).

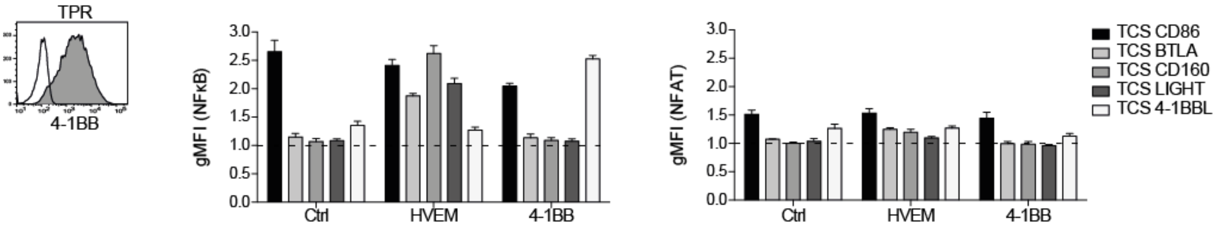

Supplementary Figure 5: Control triple parameter cells (TPR) and HVEM and 4-1BB expressing TPR were stimulated with TCS ctrl, TCS CD86, TCS CD160, TCS LIGHT and TCS 41-BBL. Reporter activation (NFAT::eGFP and NFκB::eCFP) was assessed via flow cytometry. Data of three independent experiments in duplicate is shown. Normalized reporter activation is shown (gMFI reporter gene expression induced by the indicated TCS/gMFI reporter gene expression induced by TCS-ctrl stimulated cells). Cell surface expression of 4-1BB on control TPR (open histogram) and 4-1BB expressing TPR (grey histogram) is shown. + SD is shown. Dotted line depicts control stimulation

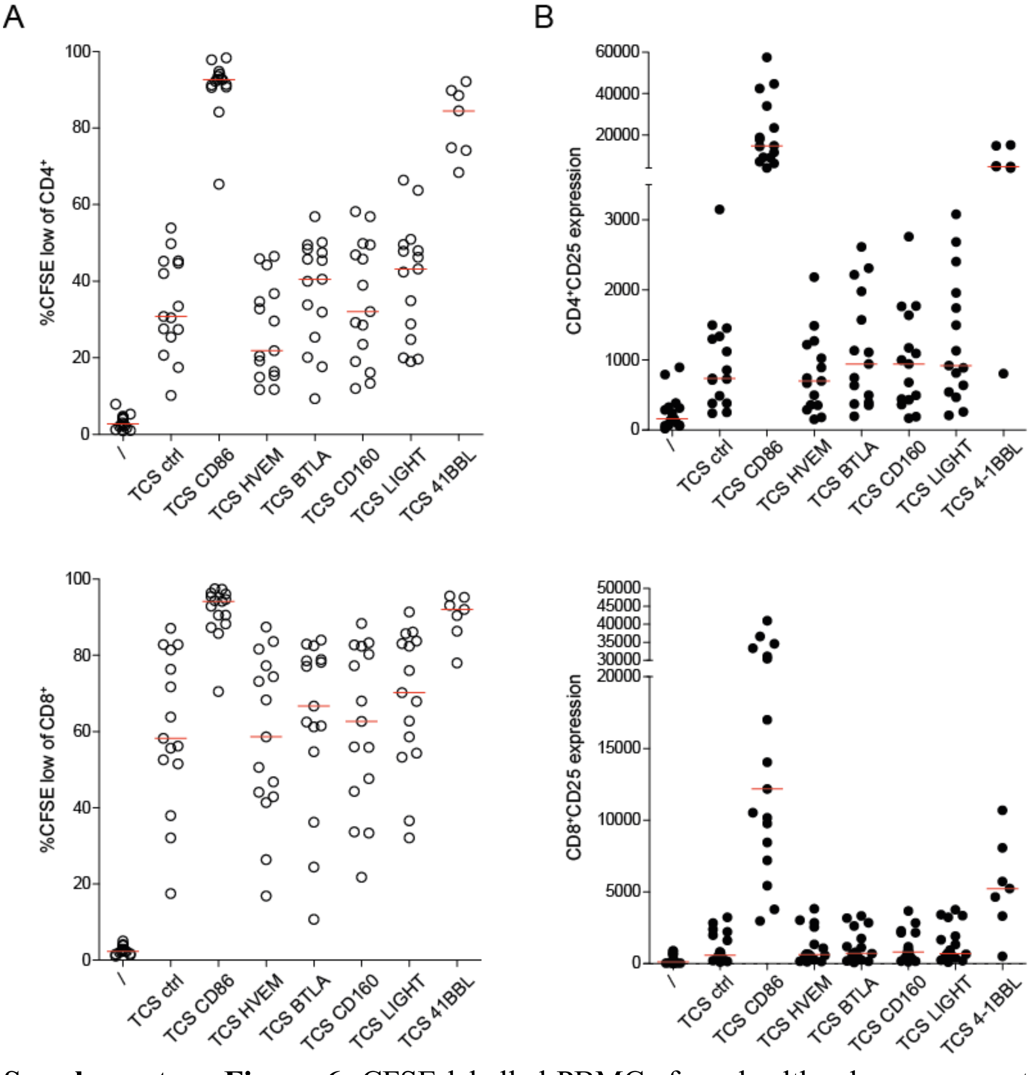

Supplementary Figure 6: CFSE-labelled PBMCs from healthy donors were stimulated for 5 days with the indicated TCS cells. Proliferation (CFSE^{low}) and activation (CD25 expression) of CD4^{+} and CD8^{+} T cells was measured at day 5. Each data point represents the mean of triplicate measurement of one donor (n = 15; TCS 4-1BBL n = 7). Median is shown.

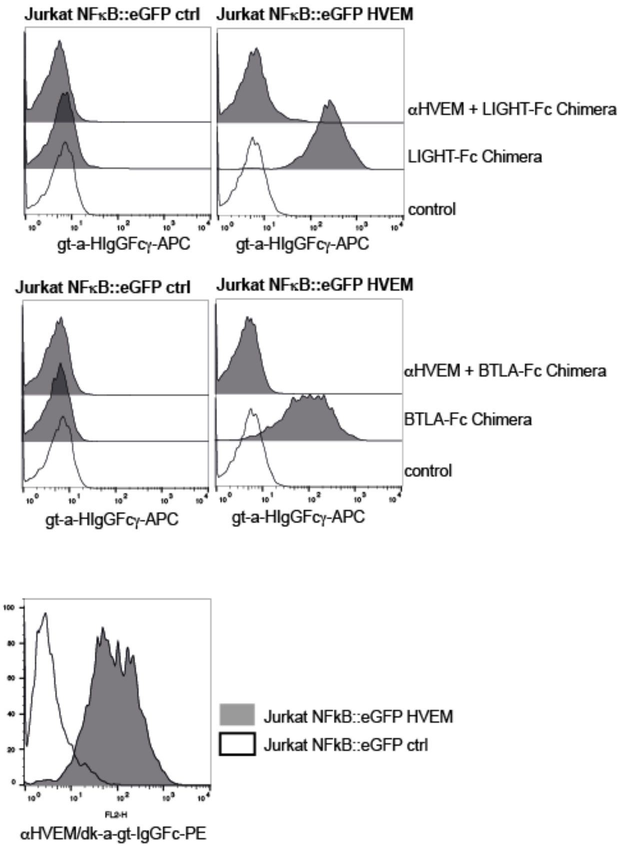

Supplementary Figure 7: Evaluation of polyclonal HVEM antibody AF356. For blocking assays Jurkat-NF-κB::eGFP control and HVEM expressing cells were incubated with the polyclonal HVEM Ab AF356 (final 10 μg/ml) for 20 minutes at 4°C. After a washing step, LIGHT-Fc or BTLA-Fc fusionproteins (final 3 μg/ml) was added. (control: detection antibody only). Binding was detected with goat-anti human IgG-Fcγ antibodies (Jackson ImmunoResearch). Lower panel depicts staining of Jurkat-reporter control cells and reporter cells expressing HVEM with the polyclonal HVEM antibody AF356 used for the blocking assays shown above.

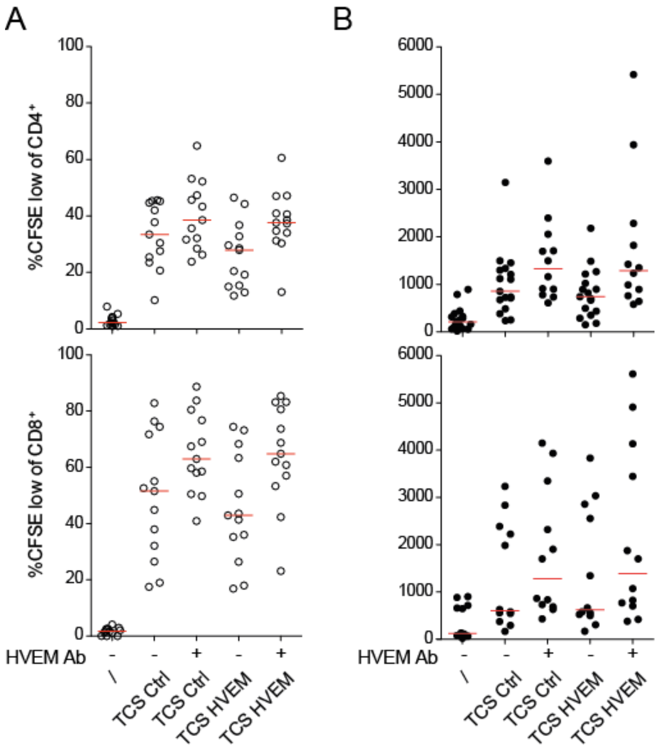

Supplementary Figure 8: CFSE-labelled PBMCs from healthy donors were stimulated for 5 days with the indicated TCS cells. Proliferation ($CFSE^{low}$) and activation (CD25 expression) of $CD4^{+}$ and $CD8^{+}$ T cell subsets was measured at day 5. Each data point represents the mean of triplicate measurement of one donor (n=12). Median is shown.

Reagent	Clone	Labeling	Dilution (final)	Source
**Mouse IgG1, k, Isotype ctrl**	MPOC-21	PE/APC	1:200	Biolegend (San Diego,CA)
**BTLA**	MIH26	PE/APC	1:300	Biolegend (San Diego,CA)
**HVEM**	122	PE	1:300	Biolegend (San Diego,CA)
**CD28**	CD28.2	PE	1:300	Biolegend (San Diego,CA)
**CD86**	IT2.2	PE	1:500	Biolegend (San Diego,CA)
**CD83**	HB15e	PE	1:600	Biolegend (San Diego,CA)
**CD160**	BY55	PE	1:200	Biolegend (San Diego,CA)
**LIGHT**	T5-39	PE/APC	1:300	Biolegend (San Diego,CA)
**mHVEM**	HMHV-1B18	PE	1:300	Biolegend (San Diego,CA)
**CD14**	63D3	APC	1:100	Biolegend (San Diego,CA)
**CD56**	HCD56	FITC	1:400	Biolegend (San Diego,CA)
**CD19**	HIB19	BV421	1:400	Biolegend (San Diego,CA)
**CD4**	OKT4	BV421	1:400	Biolegend (San Diego,CA)
**CD8**	HIT8α	PerCP	1:400	Biolegend (San Diego,CA)
**CD25**	M-A251	PeCy7	1:600	Biolegend (San Diego,CA)
**ICOS**	C398.4a	APC	1:600	Biolegend (San Diego,CA)
**mCD45.2**	#104	APC	1:800	Biolegend (San Diego,CA)
**HVEM**	SL030717	/	*	DOI: 10.1182/blood-2012-11-464685
**HVEM**	polyclonal AF365	/	*	R&D systems (Minneapolis,MN)
**CD160**	688327	/	1:100	R&D systems (Minneapolis,MN)
**BTLA**	6F4	/	5 µg/ml	Adipogen Life Sciences (San Diego, CA)
**Goat anti-mouse IgG (Fc specific)**	/	PE	1:300	Jackson ImmunoResearch, West Grove, PA)
**Goat-anti-mouse IgG-H+L**	/	PE	1:200	Jackson ImmunoResearch, West Grove, PA)
**Goat-anti-human IgG Fcγ**	/	APC	1:200	Jackson ImmunoResearch, West Grove, PA)
**Human LIGHT-Fc-Tag (active Trimer)**	/	/	3 µg/ml	AcroBiosystems
**Human BTLA-Fc Chimera**	/	/	3 µg/ml	Biolegend (San Diego,CA)

Supplementary Table 1: Antibodies and fusionproteins used in this study.
* used at different concentrations in functional reporter and primary cell assays, respectively (Figure 2, figure 4, figure 7 and Supp. Figure 7)
